# Anti-Spike SARS-CoV-2 IgG Assessment with a Commercial Assay during a 4-Month Course after COVID-19 Vaccination

**DOI:** 10.3390/vaccines9111367

**Published:** 2021-11-20

**Authors:** Jakub Swadźba, Tomasz Anyszek, Andrzej Panek, Emilia Martin

**Affiliations:** 1 Department of Laboratory Medicine, Faculty of Medicine and Health Sciences, Andrzej Frycz Modrzewski Krakow University, 30-705 Krakow, Poland; jakub.swadzba@diag.pl (J.S.); tomasz.anyszek@diag.pl (T.A.); 2 Medical Department Diagnostyka S.A., 31-864 Krakow, Poland; andrzej.panek@diag.pl

**Keywords:** SARS-CoV-2 antibodies, COVID-19 testing, COVID-19 vaccines, COVID-19 vaccines booster shot

## Abstract

We intended to assess the humoral response induced by the Pfizer/BioNTech Comirnaty COVID-19 vaccine with commercially available immunoassays: anti-spike (S) IgG and IgM, and anti-nucleocapsid (N) IgG antibodies, over a 4-month course. One hundred subjects, including 15 COVID-19 convalescents, comprised the study cohort. The SARS-CoV-2 antibodies concentrations were measured on day 0′ and 10′, 20′, 30′, 60′, 90′, and 120′ after the first dose administration. Over the course of the study, 100% of the participants developed and sustained anti-SARS-CoV-2 S IgG antibodies. The highest concentration, exceeding the quantification range of the test (2080 BAU/mL), was reached by 67% of the subjects on day 30′. The concentration of the antibodies remained stable between days 30′ and 90′ but was followed by a significant decrease between days 90′ and 120′. The stronger and more persistent humoral response was noted for women. The COVID-19 convalescents developed higher antibody levels, particularly 10 days after the first Comirnaty dose. Twenty-three out of the eighty-five naïve vaccinees failed to develop a detectable IgM response. LIAISON^®^ SARS-CoV-2 TrimericS IgG (DiaSorin S.p.A, Saluggia, Italy) may be useful in the assessment of the humoral response to the Comirnaty vaccine. In contrast, Abbott’s anti-S SARS-CoV-2 IgM has a limited utility in this context.

## 1. Introduction

Considering the scarcity of an available effective COVID-19 treatment [1], the vaccinations have become the focal point of the efforts to hamper the pandemic. The COVID-19 vaccines have been developed at an unprecedented speed, with the first being endorsed within less than a year from the identification of the infectious agent: severe acute respiratory syndrome coronavirus 2 (SARS-CoV-2). The attitude towards vaccination varies, and many people are hesitant about getting inoculated [2], which is likely to curb the achievement of the herd immunity. Studies show that people with a higher level of knowledge of COVID-19 vaccines are more likely to get vaccinated [3]. Apart from the data from the clinical trials, the real-world observations may contribute to the public’s knowledge and, hence, a positive approach towards the COVID-19 vaccination. One such observation may be the proof of the immunogenicity of the vaccines—high levels of specific antibodies—obtained by a simple, commercially available blood test.

The serological assays detecting SARS-CoV-2 antibodies have evolved dramatically since the beginning of the pandemic. The lateral flow chromatography assays (LFIAs) were the first available, allowing for a qualitative assessment of IgG and IgM antibodies [4]. The design of these simple cassette tests initially employed a mixture of undefined SARS-CoV-2 antigens. Before long, the attention switched to the automated immunochemical tests: enzyme-linked immunosorbent assays (ELISAs) and chemiluminescent immunoassays (CLIAs) [5]. These methods allowed for the semi-quantitative or quantitative measurement of IgA, IgM, and IgG antibodies solely against the nucleocapsid (N) antigen or against spike (S) antigen, and, in some tests, both antigens [6,7,8]. It was also recognized that the antigenic specificity of the antibodies is an important feature, influencing the clinical specificity and sensitivity of the assays [9,10,11,12,13]. The anti-SARS-CoV-2 antibodies recognizing the S protein of the virus, especially its receptor binding domain (RBD), were proven to be of the utmost importance for the virus neutralization capacity [14]. All four vaccines approved currently in the EU are designed to induce an immune response against the SARS-CoV-2 spike protein.

The importance of the serological testing in the immune response against SARS-CoV-2 manifests in two fields: natural infection and vaccination. For the latter, the tests using antigen S should only be used. Natural infection in vaccinated subjects may only be discerned with the tests based on the N antigen.

The aim of this study was to assess the humoral response induced by the vaccination over a 4-month course following the administration of the first dose of the Pfizer–BioNTech COVID-19 vaccine (Comirnaty). 

Observations of this nature may guide the future vaccination strategies, including those directed at the identification of the non-responders and those verifying the necessity of the administration of a third dose. 

## 2. Materials and Methods

### 2.1. Study Design

The study participants (*n* = 100) were recruited from professionally active healthcare workers vaccinated against COVID-19 between 4 January 2021 and 26 March 2021 in Cracow, Poland. The subjects were selected randomly, with no exclusion criteria. All subjects received Pfizer–BioNTech Comirnaty vaccine (BioNTech Manufacturing GmbH, Mainz, Germany), with two doses administered 21 days apart. The cohort included one hundred subjects, 86% females and 14% males. The mean age of the study group was 45 (23–74), and there was no statistically significant difference in age between the sexes. There were 89 subjects under 60 years old.

The blood for antibodies assessment was drawn on day 10′, 20′, 30′, 60′, 90′, and 120′ after the first dose of the vaccine. For 67 subjects, the samples were obtained on the day of the vaccination (0′) as well.

All of the subjects provided informed consent for the participation in the study and filled out a questionnaire about their history of COVID-19.

### 2.2. Laboratory Testing

The LIAISON^®^ SARS-CoV-2 TrimericS IgG (DiaSorin S.p.A, Saluggia, Italy) immunoassay was performed on fresh blood sera on the day of blood collection, and the remaining sera were aliquoted and frozen (−20 °C) for additional testing. The testing was performed on the LIAISON^®^ XL analyzer strictly according to the manufacturer’s instructions. This assay allows for a quantitative measurement of IgG class antibodies against a trimeric S-protein. The quantification is based on the chemiluminescent signal that is induced at the final stage of the procedure and measured in relative light units (RLU). The reading is proportional to the concentration of the antibodies in the sample, which is expressed in arbitrary units (AU/mL). Owing to the correlation of the results of LIAISON^®^ SARS-CoV-2 TrimericS IgG to the values and units of the first WHO International Standard (IS) for anti-SARS-CoV-2 immunoglobulin binding activity (NIBSC 20–136) [15], the AU/mL may be converted to the binding antibody units (BAU/mL) through a multiplication by a factor of 2.6. The assay’s quantification range is between 4.81 and 2080 BAU/mL, and a cut-off for a positive result is 33.8 BAU/mL. The samples for which the results exceeded 2080 BAU/mL were diluted 1:20, according to the manufacturer’s instructions, and re-tested. The producer had correlated the LIAISON^®^ SARS-CoV-2 TrimericS IgG results ≥520 BAU/mL with a high neutralization capability in the microneutralization assay. Hence, the presence of this type of antibodies may be considered one of the correlates of immunity.

The additional testing was conducted on the remnant serum samples frozen on the day of blood collection. Anti-SARS-CoV-2 spike-specific IgM antibodies were assessed to provide a full picture of the seroconversion induced by the vaccination. Nucleocapsid-specific IgG antibodies were tested to check for the possible unknown SARS-CoV-2 infection prior to the study, as well as for the contact with the virus over the course of the study. The testing was performed with Abbott’s SARS-CoV-2 chemiluminescent microparticle immunoassays (CMIA) on Alinity i analyzer (Abbott Ireland, Sligo, Ireland), strictly to the manufacturer’s instructions. These assays allow for a qualitative detection of the antibodies. The results are expressed as indices, calculated as a ratio of sample and calibrator signals. A cut-off for a positive result of IgM test is 1.0, and the cut-off for IgG test is 1.4.

### 2.3. Statistical Analysis

The statistical analysis was performed with STATISTICA software ver. 13 (TIBCO Software Inc., Palo Alto, CA, USA). Friedman’s average rank test was applied to assess the statistical differences in the antibody titers between the time points. Dunn’s test with Bonferroni adjustment was used as a post hoc test for Friedman’s test. Mann–Whitney U test was applied to detect the statistical differences in the antibody titers between the subgroups. Student’s *t*-test was used to verify the statistical significance of the difference in age between the sexes. The Spearman’s rank correlation coefficient was used to assess the correlation between the age and the level of antibodies. The significance level was set to 0.05.

## 3. Results

The history of COVID-19, confirmed by RT–PCR or antigen testing in the past 2–10 months, was reported in the questionnaire by eight subjects. Additionally, the serological status of those convalescents was checked. The day 0′ samples were available for six of the eight confirmed COVID-19 cases. Four of these six patients were positive in the LIAISON^®^ SARS-CoV-2 TrimericS IgG assay. One of the remaining convalescents was positive in both Abbott assays (anti-N IgG and anti-S IgM), and the other was positive only in anti-N IgG. In one of the cases without a 0′ sample, a positive result was obtained for anti-N IgG in day 10′ sample, but, in the other case, confirmed with a positive RT–PCR test ten months prior to the vaccination, anti-N IgG antibodies were already absent.

Additionally, a serological assessment was used to detect the possible undiagnosed COVID-19 cases. Out of the 61 subjects with no history of COVID-19 tested on the day of the first dose administration (day 0′), seven (11.47%) were seropositive in at least one of the assays. Hence, they were considered convalescents in the following analyses. For the subjects with no COVID-19 history without available day 0′ samples, we also analyzed the anti-N IgG results on day 10′. None of them were positive.

Eight cases with a history of COVID-19 and seven cases serologically positive on day 0′ constituted a group of 15 COVID-19 convalescents. 

### 3.1. LIAISON^®^ SARS-CoV-2 TrimericS IgG Results

#### 3.1.1. LIAISON^®^ SARS-CoV-2 TrimericS IgG Results in the Whole Cohort

Over the course of the study, 100% of the participants developed and sustained a measurable concentration of anti-SARS-CoV-2 trimeric S IgG antibodies. 

**Figure 1:**
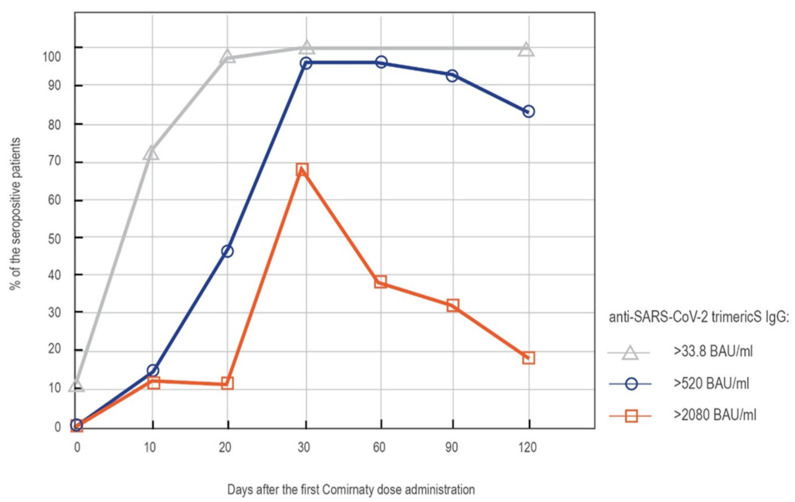
The percentage of the patients reaching given antibody levels at the consecutive time points after vaccination. Results obtained with LIAISON^®^ SARS-CoV-2 TrimericS IgG assay; >33.8 BAU/mL—cut-off for the positive result; >520 BAU/mL—antibody concentration correlated with a high neutralization capability; >2080 BAU/mL—upper limit of the assay’s quantification range.

The seropositivity rates increased at the consecutive time points—from 73% on day 10′ to 97% on day 20′ (just before the second dose)—and reached 100% on day 30′ (almost 10 days after the second dose of the vaccine). All the subjects remained seropositive until the end of this study—four months after the first vaccine dose administration. The highest concentration of anti-S IgG antibodies, exceeding the quantification range of the test (2080 BAU/mL), was reached by the majority of the subjects (67%) on day 30′. The number of patients with an extremely high level of antibodies gradually declined after that time point. However, the level of antibodies higher than 520 BAU/mL, correlated by the producer with a high neutralization capability, was still observed in 83% of the patients on day 120′ (Figure 1).

**Figure 2:**
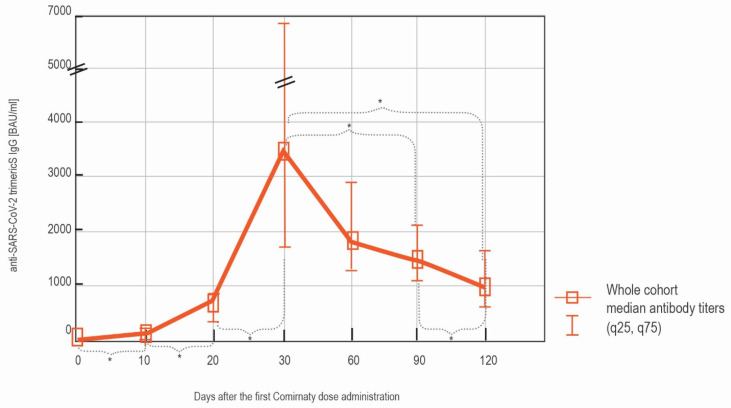
The serodynamics of the anti-spike IgG production after vaccination in the whole cohort. The results obtained with LIAISON^®^ SARS-CoV-2 TrimericS IgG assay at each of the time points are shown as median (q25, q75). The significant differences between the measurements (*p* < 0.05) at the consecutive time points are indicated with *.

One-hundred eighty-three samples with the results exceeding the upper limit of quantification (ULQ) of the assay, 2080 BAU/mL, were diluted 1:20 according to the manufacturer’s instructions and re-tested. Eighty-seven of the obtained results were lower than two times the ULQ (4160 BAU/mL), fifty-one were between two and four times the ULQ (up to 8320 BAU/mL), thirty-three between four and ten times, and seven above 20,800 BAU/mL (>10 times). The statistical analysis of the differences between the antibodies levels at the consecutive time points was performed. A statistically significant increase was observed between day 0′ and day 10′, day 10′ and day 20′, which corresponds to the first dose administration, as well as between day 20′ and day 30′—after the second dose. The concentration of the antibodies decreased insignificantly between day 30′, 60′, and 90′ but was followed by a significant drop between day 90′ and day 120′ (Figure 2).

#### 3.1.2. LIAISON^®^ SARS-CoV-2 TrimericS IgG Results in in Convalescents and Non-Convalescents

COVID-19 convalescents developed higher antibody concentrations than patients with no history of this disease at all of the studied time points. The difference between the antibody levels was statistically insignificant only on day 30′ (Table 1). Between days 10′ and 30′, a constant rise in the antibody concentration was noted in the naïve vaccinees, whereas, in the convalescent group, the level was already decreasing.

Particularly pronounced was the difference in the amount of the antibodies on day 10′. At this time point, 86.7% (13 out of 15) of the convalescents reached the upper quantification limit of the test (2080 BAU/mL). Such a value was reported in none of the non-convalescents on day 10′.

**Figure 3:**
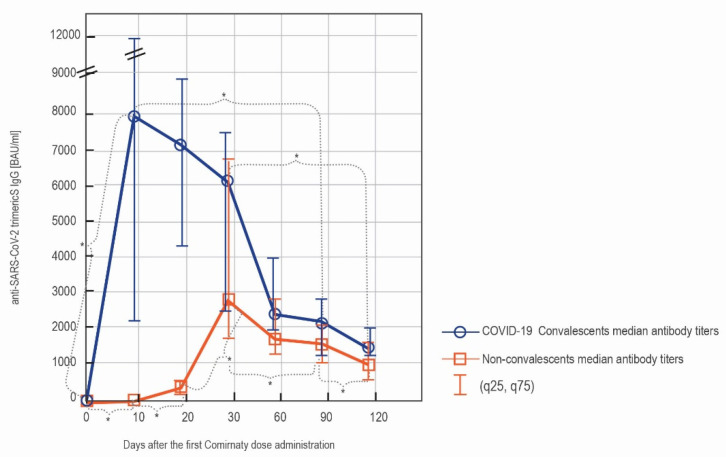
The comparison of the serodynamics of the anti-spike IgG production in the subgroup of COVID-19 convalescents (blue) and naïve vaccinees (orange). The results were obtained with LIAISON^®^ SARS-CoV-2 TrimericS IgG and are shown as median (q25, q75). The significant differences (*p* < 0.05) between the measurements at the consecutive time points are indicated with *.

The first dose administration caused in COVID-19 convalescents a sharp and statistically significant increase in the antibody concentrations between day 0′ and day 10′. Afterwards, the levels of antibodies in the convalescents remained at a high level, with no statistically significant differences between the antibody concentrations at the consecutive time points (10′–20′–30′–60′–90–120′). However, when the antibody titers peaking after the first vaccine dose administration were compared with the furthest time points of the study (day 90′ and day 120′), the decline in the concentrations was significant. In non-convalescents, a statistically significant increase was seen between days 0′, 10′, 20′, and 30′. Similar to the convalescents, there was a significant drop between the peak of the humoral response (day 30′) and time points 90′ and 120′. There were no significant changes between the consecutive days 30′, 60′, and 90′ (insignificant decrease), whereas, in contrast to the COVID-19 convalescents, a significant decrease of the antibody levels was observed between day 90′ and 120′ (Figure 3).

#### 3.1.3. LIAISON^®^ SARS-CoV-2 TrimericS IgG Results in Females vs. Males

Due to the observed differences between the COVID-19 convalescents and naïve vaccinees, the convalescents were excluded from the analyses regarding the influence of sex and age on the concentration of anti-S SARS-CoV-2 IgG antibodies.

The median antibody concentrations differed between the sexes, with females developing higher titers at each of the time points, except for day 30′. The differences were, however, statistically significant only from day 60′ (Table 2).

On day 10′, almost 74% of the women and only 38% of the men were seropositive. After 4 months, the concentration of antibodies higher than 520 BAU/mL was observed in 84% of women and only 61% of men (Table 2).

**Figure 4:**
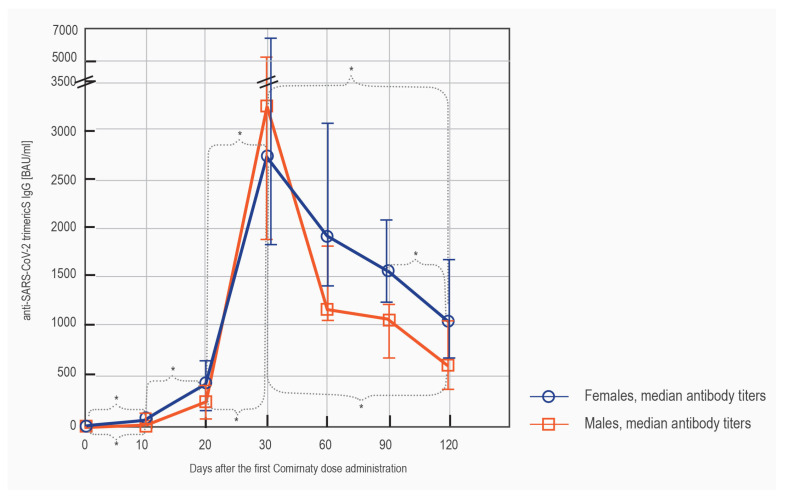
The comparison of the serodynamics of the anti-spike IgG production in the subgroups of non-convalescent females (blue) and males (orange). The results were obtained with LIAISON^®^ SARS-CoV-2 TrimericS IgG assay and are shown as median (q25, q75). The significant differences (*p* < 0.05) between the measurements at the consecutive time points are indicated with *.

The females tended to respond to the vaccination quicker than the males, with a significant increase in the antibody titer observed already between day 10′ and 20′. For both sexes, the statistically significant decrease in the antibody concentrations was seen between day 30′ and day 120′ (Figure 4).

#### 3.1.4. LIAISON^®^ SARS-CoV-2 TrimericS IgG Results in Subjects below and over 60 y/o

The younger subjects (below 60 y/o) displayed higher antibody titers over the first month after the vaccination. However, the differences observed were only significant on day 30′. After that time point, there were no statistically significant differences between the younger and older vaccinees in the antibody titers, with the median antibody titers observed in vaccinees >60 y/o being actually slightly higher than in younger subjects. (Table 3). 

**Figure 5:**
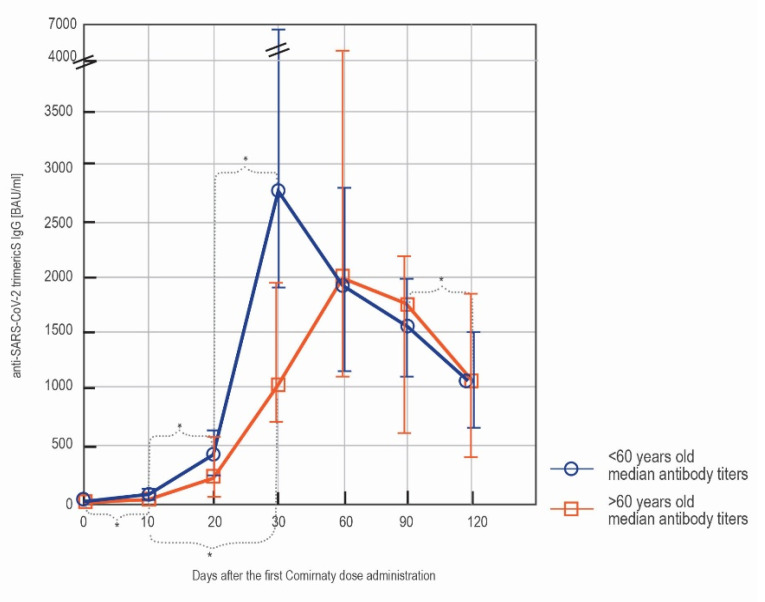
The comparison of the serodynamics of the anti-spike IgG production in the subgroup of non-convalescent subjects below 60 y/o (blue) and over 60 y/o (orange). The results were obtained with LIAISON^®^ SARS-CoV-2 TrimericS IgG assay and are shown as median (q25, q75). The significant differences (*p* < 0.05) between the measurements at the consecutive time points are indicated with *.

The dynamics of the humoral response to the vaccination were influenced by the age of the subjects. The younger ( <60 y/o) vaccinees quickly mounted a strong response that was muted shortly afterwards. The antibody production in the older ( >60 y/o) vaccinees was less dynamic: the antibody titer increased gradually to reach titers similar to the younger subjects on day 60′. No statistically significant decrease in the antibody titer in this subgroup was seen over the course of 4 months. In the younger subjects, the decrease between day 90′ and 120′ was statistically significant (Figure 5).

**Figure 6:**
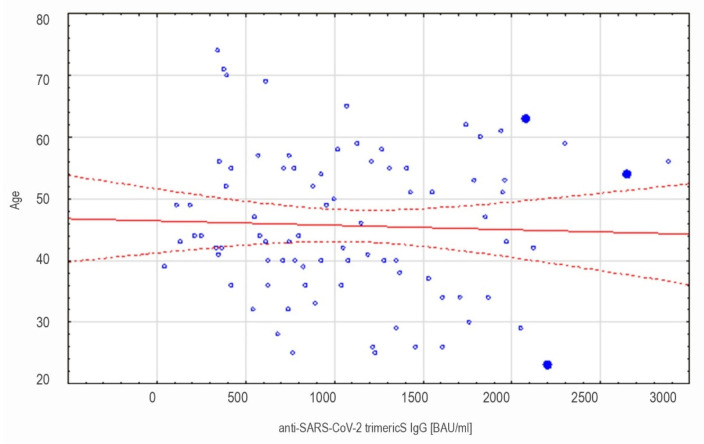
The scatter plot showing association between age and antibody level 120 days after vaccination in non-COVID-19-convalescents. The results obtained with LIAISON^®^ SARS-CoV-2 TrimericS IgG assay. No correlation was found (r = −0.045).

In the naïve vaccinees, a correlation between the antibody concentration and age was assessed on day 120′. No association between these two variables was found (r = −0.045 *p* = 0.685) (Figure 6). 

### 3.2. Alinity SARS-CoV-2 S IgM Testing Results

**Figure 7:**
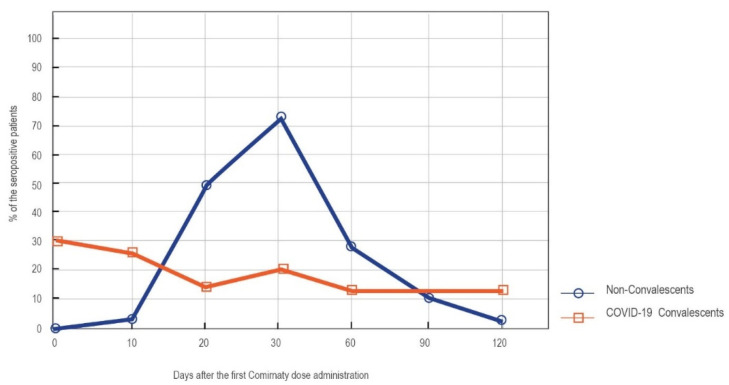
The percentage of the patients reaching a cut-off for a positive result of anti-S IgM antibodies at the consecutive time points after vaccination. Results obtained with SARS-CoV-2 S IgM Abbott assay.

Early phase IgM class antibodies recognizing the spike protein of SARS-CoV-2 are induced by both natural infection and vaccination. In our study, positive results of this type of antibodies on day 0′ were only observed for COVID-19 convalescents (four cases). Starting from day 10′, these antibodies began to be detectable in the vaccinated subjects without a prior infection. However, at none of the time points studied did the seropositivity rates reach 100%. The percentage of the convalescents showing positive IgM titers did not increase significantly at any point over the course of the study, whereas the seropositivity rates in the naïve vaccinated subjects increased during the first month after the vaccination and decreased later. Of note, 23 out of the 85 subjects without a prior SARS-CoV-2 contact failed to develop any detectable IgM response, and only 72.84% were seropositive for anti-S IgM antibodies at the peak of the vaccination-induced response (day 30′). On day 120′, anti-S IgM antibodies were only detected in two out of the eighty-five naïve vaccinees (Figure 7).

### 3.3. SARS-CoV-2 N IgG Testing Results

Anti-nucleocapsid (N) SARS-CoV-2 antibodies can only be detected after a natural infection. Hence, the seropositivity rates obtained in this assay in our study were low and observed, as expected, in some of the COVID-19 convalescents (eight out of fifteen). It should be outlined that, in the RT–PCR or antigen-confirmed COVID-19 convalescents, anti-N IgG antibodies were not detected over 4 months after the diagnosis. During the study, the declining rate of this type of antibodies was seen in all cases.

Over the course of the study, anti-N IgG antibodies were also discovered in four fully vaccinated subjects. Two of them started to be positive on day 60′, one on day 90′, and one on day 120′ (Table 4). Most probably, they were in contact with the virus. In two of those cases, there was a noticeable increase in the level of anti-S IgG antibodies, concomitant to the emergence of anti-N antibodies. This indicates that a SARS-CoV-2 encounter in the vaccinated subjects may act as a booster of the immune response.

Taking into consideration the possible confounding influence of the infection on the results of this study, we excluded those four patients from the cohort and re-analyzed all the results. No change in the statistical significance of the results was noticed (data not shown).

## 4. Discussion

The serological testing proved useful in both natural disease recognition and in vaccine immunogenicity studies. Using serology in the context of the COVID-19 vaccination has been a subject of experts’ scrutiny for a few months [16,17]. The main questions raised concern the type of antibodies that should be assessed, the assays to be used, and the time points at which the testing should be performed. The last involves both the time points at which non-responders may be detected, as well as those when the need for a booster should be considered. Follow-up questions involve the correlates of immunity: could specific antibody titer be one of them, and, if so, what values could be considered protective?

Our study was not aimed at providing the definite answers to the above questions. However, observations like ours add to the public’s knowledge on the immune response to the immunization and may help to optimize the vaccination strategies.

This study provides evidence for a robust humoral response to the Pfizer–BioNTech Comirnaty vaccine that may be detected and quantified by a commercially available immunoassay. We showed that 100% of the participants were seropositive for anti-trimeric S SARS-CoV-2 IgG antibodies within 30 days from the first vaccine dose administration (nine days after the second dose) and, in all the subjects, these antibodies were still detectable on day 120′. The dynamics of the antibody production differed between the COVID-19 convalescents and the participants without a previous SARS-CoV-2 infection. Similar to Terpos et al. [18] and Manisty et al. [19], we proved that the first dose of the vaccine acts in the convalescents as a strong booster of an already existing humoral response and manifests by extremely high antibodies concentrations as early as 10 days after the first dose administration. A similar increase was seen in the non-convalescents only after the second dose, i.e., on day 30′. Of note, this phenomenon was seen in both swab-confirmed and serologically confirmed convalescents. This is contrary to the results of the study of Demonbreun et al. [20], who reported significantly stronger responses to the first dose of COVID-19 vaccination in convalescents with a history of acute viral testing (*n* = 46) in comparison to those who were solely baseline seropositive (*n* = 144). This difference may be caused by the small size of our study group and requires further elucidation. Our study was of the observational type, so the convalescents underwent the vaccination according to the guidelines and received a second dose. We did not observe a significant increase in the antibody concentrations in the convalescents after the second dose. On the contrary, the antibody level reached its peak on day 10′ and gradually declined after that time point. Similar findings had been reported in a pre-print study [21]: a robust humoral response after the first dose followed by a muted reaction after the second administration of an mRNA COVID-19 vaccine in SARS-CoV-2-experienced individuals. Salvagno et al. [22] observed only a modest rise in the antibody levels in the convalescents after the second dose. The above observations may be a preliminary indication that the booster dose may be, if not neglectable, then at least delayed in time in COVID-19 convalescents. This has already been considered and commented on by, e.g., Focosi et al. [23]; however, further research directed at that issue will be needed. Some data on the relationship between the response to the COVID-19 vaccination in convalescents and factors such as the baseline antibody level and symptoms developed over the disease course were provided by Levi et al. [24]. Those researchers concluded that a single dose may be sufficient in certain groups of COVID-19 convalescents.

An important observation from our study is also a strong decline of the antibody levels 4 months after the first dose administration. This supplements and particularizes the findings of Tré-Hardy et al., who reported a significant antibody decline between the measurements 3 and 6 months after the first dose of the mRNA vaccine administration [25]. Based on Tré-Hardy’s and similar findings, Lippi et al. suggested that the antibody titration in the context of the COVID-19 vaccination could be performed at the baseline, after a month, and then after 6 months [16].

In agreement with a few previously published papers [18,22,26,27,28], we found that the antibody concentrations depended on the sex and age of the subjects. For example, Lo Sasso et al. reported significantly higher basal post-vaccination anti-S-RBD IgG levels in females in comparison to males but no significant difference in the rate of the antibody decay [27]. In contrast to that, we observed similar levels of the antibodies at the peak of the response (day 30′), with the differences between the sexes revealing themselves afterwards. In our study, the higher level of the antibodies in women versus men was particularly pronounced on days 60′, 90′, and 120′, which could indicate a more sustained IgG production in women.

It has also been reported [26,27,28,29] that the antibody production is affected by the age of the subjects. Our results picture a similar tendency, but only over the first month of the study, when the younger subjects (<60 y/o) produced more antibodies. However, the differences observed were significant only on day 30′, when younger participants reached the maximum median concentration (3360 BAU/mL). The older subjects mounted the strongest response later, on day 60′ (median concentration of 2000 BAU/mL). This delay in the immune reaction in the elderly was also noted by Schwarz et al. [28]. However, we did not observe a correlation between the age and anti-spike IgG concentration four months after vaccination. Together, these findings may indicate that, although it takes longer for the elderly to develop the optimal response to the vaccination, once it is achieved, the antibody concentrations are similar to the younger vaccinees.

It may be assumed that the immunity to SARS-CoV-2 infection is at least to some extent dependent on the specific binding and/or neutralizing antibodies. One of the studies on mRNA COVID-19 vaccine effectiveness in the real-world setting reported decreasing yet still strong protection against infection in vaccinees followed for 5 months [30]. This seems to be reflected in the antibody titer waning observed over the course of our study, which was, however, still high in the majority of the subjects 4 months after the vaccination. The information or even clues on the protective antibody concentration are, unfortunately, still lacking. Hence, the surrogate indicators tend to be used to provide some information on the level of protection associated with a given antibody concentration. One such indicator is the correlation between the anti-S antibody level and the neutralization capability. Since the SARS-CoV-2 spike (S) glycoprotein promotes the entry into host cells [14], the antibodies recognizing this protein, or its subunit, RBD, have been considered neutralizing. The assay used in this study quantifies the antibodies targeting the trimeric structure of the spike protein. This test design widens the spectrum of the exact antibodies specificities that are detected and may be related to the overall neutralization capability. Based on the producer’s validation studies, the results higher than 520 BAU/mL, obtained with the LIAISON^®^ SARS-CoV-2 TrimericS IgG, could be considered protective. Such a value was reached at least at one time point of the study by all of the participants. Acknowledging this optimistic finding, we must mention that, on day 120′, 17 subjects had antibody concentrations below this threshold. Of note, five of them were male (more than 1/3^rd^ of the men included in this study), which further points to the weaker humoral response in men.

The studies performed thus far concentrate on the assessment of anti-spike IgG antibodies after COVID-19 vaccination. Our study provides some insights into the formation of anti-spike IgM antibodies after vaccination. In the COVID-19 convalescents, no response in IgM class antibodies was seen after the first and second dose administration. Clearly, this is related to the immunological memory phenomenon that leads to the direct and swift production of IgG class antibodies following the pathogen re-encounter. In the naïve vaccinees, an increase in the IgM anti-S antibody levels was seen after the vaccine administrations and reached the peak on day 30′ after the first dose. However, a sharp decrease was noted afterwards, and IgM were practically not detected after 3–4 months. Importantly, not all of the naïve vaccinees mounted a detectable IgM response. In the context of an extremely strong and specific immunization during vaccination, it may be suspected that the immune response is shifted instantly towards the more efficient IgG antibodies. Hence, in our opinion, the response to the vaccination should not be monitored with the IgM class antibodies.

The results obtained in our study show that an anti-N response may be mounted, presumably following a SARS-CoV-2 encounter, in COVID-19 fully vaccinated individuals. This proves that, for the purpose of the epidemiological studies, even in the vaccinated population, assays detecting anti-N IgG antibodies may still be used, provided that their limitations are considered.

Our study is not devoid of limitations. The subgroups of the COVID-19 convalescents, males, and subjects over 60 y/o were underrepresented, which may have affected the significance levels observed in this study. Moreover, the samples for which the results of the anti-trimeric SARS-CoV-2 spike IgG assay exceeded the upper quantification limit of the test were diluted after being stored in a freezer (−20 °C) for a few months, which could have affected the samples’ stability and falsely decreased the results obtained. 

Nonetheless, the results obtained support and add to the increasing body of evidence justifying the use of serology testing throughout the vaccination course. As we showed above, the basal level of the antibody may be used as a predictor of the response to the vaccination and possibly allow for an informed delay of the vaccination in certain cases. A significant decrease observed in our study between the measurements on days 90′ and 120′ indicates that serology testing may be useful, especially 120 days after vaccination. We are aware that the antibodies are just one of the indicators of the immunity to COVID-19 and the observed drop in their levels does not need to imply the lesser protection against anti-SARS-CoV-2. Future studies should, therefore, be aimed at establishing a correlation between the antibody levels, ideally complemented with the detection of the T-cell SARS-CoV-2-specific reactivity [31], and the COVID-19 incidence and severity.

## Figures and Tables

**Table 1:** SARS-CoV-2 TrimericS IgG over 4 months course after COVID-19 vaccination in convalescents vs. naïve vaccinees.

	Naïve Vaccinees vs. COVID-19 Convalescents						
Days after the First Comirnaty Dose Administration	Prior SARS-CoV-2 Contact	Number of Subjects	% of the Subjects Reaching a Given Antibody Concentration			Median Antibody Concentration	
			>33.8 BAU/mL	>520 BAU/mL	>2080 BAU/mL	BAU/mL	*p*
0′	Naïve vaccinees	54	0	0	0	4.81	*p* = 0.000
	COVID-19 convalescents	13	61.5	0	0	41.5 *	
10′	Naïve vaccinees	85	68.2	1.2	0	67	*p* = 0.000
	COVID-19 convalescents	15	100	93.3	86.7	7945 *	
20′	Naïve vaccinees	85	96.5	38.8	0	408	*p* = 0.000
	COVID-19 convalescents	15	100	93.3	80.0	7180 *	
30′	Naïve vaccinees	85	100	96.5	65.9	2865	*p* = 0.175
	COVID-19 convalescents	15	100	93.3	80.0	6165	
60′	Naïve vaccinees	85	100	95.3	34.1	1776	*p* = 0.047
	COVID-19 convalescents	15	100	100	66.7	2325 *	
90′	Naïve vaccinees	85	100	91.8	27.1	1522	*p* = 0.022
	COVID-19 convalescents	15	100	100	66.7	2165 *	
120′	Naïve vaccinees	85	100	81.2	14.1	1010	*p* = 0.024
	COVID-19 convalescents	15	100	93.3	46.7	1485 *	

* Indicates a statistically significant difference between the median antibody concentrations at the given time points between the subgroups.

**Table 2:** SARS-CoV-2 TrimericS IgG over 4 months after COVID-19 vaccination in non-convalescent females vs. males.

	Females vs. Males *n* = 85 (72 vs. 13)					
Days after the First Comirnaty Dose Administration	Sex	% of the Subjects Reaching a Given Antibody Concentration			Median Antibody Concentration	
		>33.8 BAU/mL	>520 BAU/mL	>2080 BAU/mL	BAU/mL	*p*
10′	Females	73.6	1.4	0	76.4	0.082
	Males	38.5	0	0	15.2	
20′	Females	97.2	41.7	0	421	0.089
	Males	92.3	23.1	0	253	
30′	Females	100	95.8	66.7	2760	0.692
	Males	100	100	61.5	3260	
60′	Females	100	98.6	37.5	1932	0.017
	Males	100	76.9	15.4	1193 *	
90′	Females	100	94.4	29.2	1570	0.004
	Males	100	76.9	15.4	1089 *	
120′	Females	100	84.7	16.7	1070	0.024
	Males	100	61.5	0	615 *	

* Indicates a statistically significant difference between the median antibody concentrations at the given time points between the subgroups.

**Table 3:** SARS-CoV-2 TrimericS IgG over 4 months after COVID-19 vaccination in non-convalescent subjects below and over 60 years old.

	<60 y/o vs. >60 y/o *n* = 85 (76 vs. 9)					
Days after the First Comirnaty Dose Administration	Age Group	% of the Subjects Reaching a Given Antibody Concentration			Median Antibody Concentration	
		>33.8 BAU/mL	>520 BAU/mL	>2080 BAU/mL	BAU/mL	*p*
10′	<60 y/o	69.7	1.3	0	76.4	0.241
	>60 y/o	55.6	0	0	33.8	
20′	<60 y/o	97.4	38.2	0	416	0.241
	>60 y/o	88.9	44.4	0	222	
30′	<60 y/o	100	98.7	71.1	3360	0.009
	>60 y/o	100	77.8	22.2	1050 *	
60′	<60 y/o	100	94.7	32.9	1771	0.478
	>60 y/o	100	100	44.4	2000	
90′	<60 y/o	100	92.1	26.3	1520	0.868
	>60 y/o	100	88.9	33.3	1755	
120′	<60 y/o	100	82.9	14.5	1000	0.885
	>60 y/o	100	66.7	11.1	1070	

* Indicates a statistically significant difference between the median antibody concentrations at the given time points between the subgroups.

**Table 4:** The dynamics of anti-N SARS-CoV-2 IgG response in all subjects (*n* = 12) with at least one positive result of SARS-CoV-2 anti-N IgG Abbott assay (cut-off: 1.4) over the course of the study.

	SARS-CoV-2 Anti-N IgG [Ratio]						
Days after the First Comirnaty Dose Administration	0′	10′	20′	30′	60′	90′	120′
CASES:							
Convalescent (confirmed 4 months prior to day 0′)	**2.44**	**2.67**	**2.69**	**2.29**	**1.74**	1.32	0.58
Convalescent (confirmed 4 months prior to day 0′)	**2.17**	**1.55**	**1.43**	1.22	0.89	0.58	0.46
Convalescent (confirmed 3 months prior to day 0′)	**2.64**	**1.98**	**1.98**	**1.82**	**1.6**	1.04	0.54
Convalescent (confirmed 2 months prior to day 0′)	**1.65**	**1.49**	**1.41**	**1.42**	0.97	0.9	0.71
Convalescent (confirmed 2 months prior to day 0′)	-	**5.01**	**4.62**	**4.28**	**3.11**	**2.11**	**1.48**
Convalescent (baseline seropositive)	**6.09**	**5.85**	**5.59**	**5.68**	**5.77**	**5.88**	**5.65**
Convalescent (baseline seropositive)	**2.11**	1.15	0.95	0.9	0.49	0.39	0.25
Convalescent (baseline seropositive)	**1.24**	**1.68**	**2.0**	**1.9**	**1.62**	**1.89**	1.11
Non-convalescent	-	0,06	0,05	0,08	**4.21**	**1.59**	**1.6**
Non-convalescent	0.03	0.03	0.03	0.04	**3.01**	**1.65**	0.27
Non-convalescent	0.02	0.02	0.01	0.02	0.02	0.02	**1.59**
Non-convalescent	0.02	0.02	0.03	0.02	0.02	**1.94**	1.25

## Data Availability

The data presented in this study are available on request from the corresponding author.
